# Tolerance evaluation and genetic relationship analysis among some economically important chestnut cultivars in Türkiye using drought-associated SSR and EST-SSR markers

**DOI:** 10.1038/s41598-023-47951-7

**Published:** 2023-11-28

**Authors:** Didem Kara, Emine Orhan

**Affiliations:** 1 Department of Agricultural Biotechnology, Graduate School of Natural and Applied Sciences, Atatürk University, 25240 Erzurum, Türkiye; 2 Department of Agricultural Biotechnology, Faculty of Agriculture, Atatürk University, 25240 Erzurum, Türkiye

**Keywords:** Agricultural genetics, Biotechnology, Plant sciences

## Abstract

The aim of this study was to evaluate drought tolerance and genetic relationships among some important chestnut cultivars for Türkiye by using drought-related genomic simple sequence repeat (SSR) markers and genic expressed sequence tag-simple sequence repeat (EST-SSR) markers. Using five SSR markers, the average number of alleles (avNa), mean heterozygosity (H_avp_) and polymorphism information content (PIC) were determined to be 9.22, 0.395 and 0.375, respectively. In addition, using eight EST-SSR markers, the values of avNa, H_avp_ and PIC were determined to be 7.75, 0.309 and 0.262, respectively. All microsatellite markers used in this study showed 100% polymorphism among chestnut cultivars. In UPGMA dendrograms obtained with both SSR and EST-SSR markers, the Erfelek and Hacıömer chestnut cultivars were determined to be the most similar cultivars. Some assessments are discussed regarding drought tolerance for specific alleles obtained from the EST-SSR markers GOT045, GOT021, GOT004, FIR094 and VIT033 in chestnut cultivars. Some preliminary results regarding drought tolerance in chestnut cultivars were obtained in our study with the help of these markers. Our study also characterized the genetic relationships among chestnut cultivars of great importance using drought-related character-specific markers.

## Introduction

Chestnut belongs to the genus *Castanea* in the family *Fagaceae*, which also includes the genera *Fagus*, *Quercus*, and *Castanopsis*. There are four major species in the genus *Castanea*: European chestnut (*Castanea sativa* Mill.), Japanese chestnut (*C. crenata* Sieb. Zucc.), Chinese chestnut (*C. mollissima* Bl.), and American chestnut (*C. dentata* Borkh.). European chestnut is distributed along the Mediterranean basin and Asia Minor^1–3^. Chestnut is an economically important species that is cultivated over large areas in Portugal, Spain, France, Greece, Italy, Türkiye and the United Kingdom. Chestnuts are thought to have survived the last ice age in a few protected areas in southern Europe, northeastern Türkiye and the Caucasus. Then, this species spread north and west throughout Europe and is thought to have arrived in Italy from Asia Minor with the Greeks^4^. After China, Spain and Bolivia, which are responsible for the majority of the world’s chestnut production (2.3 million t), Türkiye ranks fourth (76,045 tons) and exports 14% of its production^5^.

Fruit culture has played an important role in Türkiye’s history. In rural areas, fruit species such as apricot, almond, walnut, chestnut, cornelian cherry, plum, hawthorn, and rose hips are grown to a large extent from seeds, resulting in a wide range of variability^6,7^. Chestnut is a type of fruit that has been cultivated in Türkiye (Anatolia) since ancient times and the fruit quality and tree characteristics vary widely. Cultivated chestnut fruits sold in the market today vary greatly. There are productive trees that produce large chestnuts with attractive and bright colours, as well as inefficient trees that produce small and low-quality chestnuts. The existence of thousands of different chestnut types in nature, together with the variation among cultivated chestnuts, is desirable in terms of breeding^8,9^. Chestnuts are popular in Türkiye and are eaten roasted or candied. Chestnut also serves as a bee fodder plant and as a landscaping tree. There are rich native chestnut populations in Türkiye, and among those populations, there are numerous genotypes resistant to chestnut blight and ink disease. For these reasons, selection studies have been carried out on chestnut populations in various parts of Türkiye since 1975^6^.

Although chestnut grows naturally (81.9%) in forested areas in Türkiye, there are also cultivated chestnut groves (11.8%). While all growers graft chestnut trees (100%) in the Aegean and Marmara Regions (in the western and northwestern parts of Türkiye), the grafting rate is 24.6% in the Western and Central Black Sea Region and 2.1% in the Eastern Black Sea Region. Due to the high annual precipitation in the Black Sea Region (northern Türkiye), chestnuts are not irrigated. The proportions of growers who irrigate chestnuts in the Aegean and Marmara regions are 42.1% and 29.2%, respectively. Growers generally use flood irrigation (61.6%), sprinkler irrigation (10.2%) and drip irrigation (28.2%). The most preferred chestnut cultivar in the Marmara Region is Marigoule, which is known to be more tolerant to chestnut blight and drought. In the Aegean Region, the Işıklar (Şekerci) cultivar is preferred as the most common cultivar due to its compatibility with consumption and marketing (roasting and candied chestnut). The Erfelek cultivar is grown in the Western Black Sea Region^10^.

In 2000, the plant materials grafted with the Marigoule cultivar obtained from Uludağ University were planted in the trial and research garden in Samsun Province, located in the Black Sea Region in northern Türkiye. The adaptation of this cultivar to the Black Sea Region has been successful. Characteristics such as yield, quality, resistance to chestnut blight and drought resistance were determined. To propagate certified grafted plants of this cultivar, it was necessary to register the cultivar. For this purpose, a registration application was submitted in 2007 and was approved in 2010^11^.

One of the major abiotic stress factors affecting the growth and development of plants is drought^12^. According to Jaleel et al.^13^, drought stress occurs when the available water in the soil decreases and when atmospheric conditions cause a continuous loss of water. Responses to drought stress show morphological and physiological variations among plant species and even among individuals of the same species^14^ and among plant organs. The degree to which genotypes are affected by drought depends on the metabolic changes (physiological and biochemical) that the genotypes have developed under stress^15,16^. There are significant changes in the physiological and biochemical contents of plants affected by drought stress. For example, changes in osmotic regulation include a decrease in cell volume, a decrease in intercellular water content, an increase in the amount of cell components, a change in photosynthetic activity, changes in the amounts of drought-related proteins such as LEA proteins, dehydrin, aquaporin, and changes in reactive oxygen metabolism^17^.

The precipitation regime is an important factor in the distribution of chestnut, and chestnuts can grow in a wide range of rainfall conditions, such as 600–1600 mm/year^18^. Chestnut species are highly affected by dry conditions. Chestnuts can be grown without irrigation in their natural environment. However, a lack of soil moisture or drought affects chestnut trees in various ways. It manifests as a decrease in yield and fruit quality (especially fruit size) and an increase in the impact of chestnut ink disease. This gains particular importance in the dry summer months^19,20^.

Climatic changes observed in chestnut-growing areas in Europe cause sensitivity in chestnuts^21^. Moreover, in Mediterranean-type ecosystems (including Türkiye), it is predicted that climate change will cause longer summer drought periods and increase the frequency and severity of drought events^22^. Moreover, as reported in Camison et al.^21^, *C. sativa* inhabits regions with marked water availability gradients (e.g., the Iberian Peninsula and Türkiye), leading to genetically based differentiation in traits related to drought adaptation. In addition, Türkiye is among the risk group countries in terms of the possible effects of global warming, and it is estimated that Türkiye’s Mediterranean and Central Anatolian regions will be more affected by climate change in the future^23^.

Molecular markers are capable of detecting DNA polymorphisms at the level of specific loci as well as at the genome level. According to the literature, simple sequence repeat (SSR) markers, also known as microsatellites, are useful in determining heterozygosity and calculating genetic distances among closely related species. Moreover, they are suitable for determining genetic indices such as the number of effective alleles as well as the polymorphism information content (PIC) in the population. Allele number and allele frequency are measures of genetic diversity in a population. The greater the number of alleles, the greater the genetic diversity. Similarly, the closer the allele frequencies are to each other, the greater the diversity^24^. SSR markers derived from expressed sequence tags (ESTs) are increasingly used to assess genetic variation and aid in the breeding and more effective conservation of trees of different genotypes. They are developed from expressed regions of the genome with known or proposed functions. Although they have been reported to be less polymorphic than genomic SSR markers, these markers are considered superior in terms of functional diversity regarding adaptive variation and interspecies transferability^22,25,26^. To date, many studies have been carried out using microsatellite-based markers (ISSR, SSR, cpSSR, EST-SSR) in chestnut species^27–43^. In these studies, researchers have used developed SSR markers^40^ or self-developed SSR markers^27^ and a variety of methods such as genotyping^29,43^, detection of genetic relationships within and among populations^35–37,39,41,42^, detection and characterization of genetic variation^30,32,38^, and characterization of relationships between populations and geography^29,31^. Additionally, there are examples of molecular characterization studies showing that markers can be transferred among different genera^28,33^. In a study in which SSR markers were used in Türkiye, five chestnut populations (Mıhlıdere, Sivrikatran, Sarıot, Ayıgedigi and Gicikdere) from Kazdagları (Chestnut Gene Protection and Management Areas) were taken as the basis, and the level of genetic diversity was determined^34^. However, to date, there has been no study on chestnuts with molecular markers designed for a specific trait in Türkiye.

Molecular characterization studies using character-specific markers also contribute to the completion of breeding studies for the selection of suitable rootstocks and scions in a short time. In chestnut cultivation, the seeds to be used in grafting must be strong, homogeneous and have a high germination rate and must also be resistant to diseases and drought, be able to be grafted at the end of the first year and be compatible with the graft^44^. For example, in a study carried out by Kulaç and Özkuru^45^, a total of 25 well-known chestnuts, including the Erfelek, Marigoule and Bethizac cultivars, which were also used in our study, were grafted onto seedlings of Kaplandağı chestnut (the rootstock chestnut genotype) with different grafting methods. In such studies, it is possible to fulfil more than one aim by conducting both characterization analyses with character-specific markers and determinations of rootstock-scion compatibility.

The first aim of this study was to evaluate drought tolerance in some important chestnut cultivars with high commercial value in Türkiye by using drought-related genomic SSR and genic EST-SSR markers. The other aim of this study was to determine the genetic relatedness levels among chestnut cultivars with the same character-specific markers through molecular characterization.

## Results and discussion

### Genetic relationship evaluation using SSR markers

Molecular markers have the power to detect DNA polymorphisms at the specific locus level as well as at the genome level. Microsatellites are useful in determining heterozygosity and calculating genetic distances among closely related species. Moreover, they are suitable for determining genetic indices such as effective allele number as well as the PIC value in the population. Allele number and allele frequency are measures of genetic diversity in a population. The greater the number of alleles, the greater the genetic diversity. In addition, the closer the allele frequencies are to each other, the greater the diversity^24^.

The five genomic SSR markers used in our study were useful for successfully determining the genetic relationship among the eleven chestnut cultivars, and the similarities and differences among these chestnut cultivars could be determined as well. In our study, the allele size ranges (SRs) of SSR markers were determined to be 23–976 bp (CsCAT1), 19–976 bp (CsCAT3), 19–976 bp (CsCAT6), 18–977 bp (CsCAT16) and 20–976 bp (EMCs38) (Table 1). Since the number of alleles (Na) and the number of polymorphic alleles (Npa) values were found to be the same, the polymorphism (P) was 100%. For the eleven chestnut cultivars, the highest Na and Npa were found for CsCAT3 (122 alleles), while the lowest Na and Npa were found for CsCAT1 (76 alleles) (avg. 101.4 alleles for all cultivars) (Table 1). In our study**,** the average numbers of alleles (avNa) obtained from SSR markers for the eleven chestnut cultivars were 6.91 (CsCAT1), 11.09 (CsCAT3), 8.82 (CsCAT6), 9.55 (CsCAT16) and 9.73 (EMCs38) (Table 1). Other studies used the same markers but obtained different results from our study. Martin et al.^33^ studied chestnut populations using the CsCAT1, CsCAT3, CsCAT6, CsCAT16 and EMCs38 markers and obtained avNa values of 10, 22, 16, 10 and 18 and allele SR values of 174–221, 189–269, 158–196, 124–153 and 229–270 bp, respectively. Janfaza et al.^39^ found an avNa value of 2.85 using the same markers. In a study carried out in chestnuts, the Na for the CsCAT1, CsCAT6 and CsCAT16 markers were determined to be 5, 6 and 2, respectively, while allele SRs were determined to be 190–236, 182–224 and 148–148 bp, respectively^34^. According to Marinoni et al.^27^, using the SSR markers CsCAT1, CsCAT3, CsCAT6 and CsCAT16 in chestnuts, a low Na (6, 4, 3 and 4, respectively) was detected. In contrast, the Na was determined to be 27 (CsCAT3) and 11 (CsCAT16) by Fernandez-Cruz and Fernandez-Lopez^36^. In Martin et al.^33^, the level of genetic diversity was successfully determined with the help of the SSR markers CsCAT1, CsCAT3, CsCAT6, CsCAT16 and EMCs38. Unlike our study, that study used the effective allele number (Ne) for evaluation, and the highest Ne values obtained were 10.53 (CsCAT3), 9.26 (EMCs38) and 7.28 (CsCAT6). For the same markers, the Ne values in Mattioni et al.^31^ were 3.35, 4.44 and 4.04, respectively. In Fernandez-Cruz and Fernandez-Lopez^36^, the Ne values of the CsCAT3 and CsCAT16 markers were determined to be 3.90 and 3.09, respectively. In Janfaza et al.^39^, the average Ne value obtained with the help of SSR markers (not used in our study) was determined as 2.163 in chestnuts obtained from four different populations.

**Table 1:** Some parametres obtained with genomic SSR markers for eleven chestnut cultivars.

SSR loci	Locus motif	Forward/reverse primer sequences (5′ → 3′)	Ta (°C)	SR (bp)	avNa	Na	Npa	P (%)	H_avp_	PIC	MI	D
CsCAT1	(TG)	F: GAGAATGCCCACTTTTGCA	50.0	23–976	6.91	76	76	100	0.464	0.339	0.464	0.410
		R: GCTCCCTTATGGTCTCG										
CsCAT3	AG	F: CACTATTTTATCATGGACGG	51.5	19–976	11.09	122	122	100	0.442	0.345	0.442	0.435
		R: CGATTGAGAGTTCATACTC										
CsCAT6	(AC)AT(AC)	F: AGTGCTCGTGGTCAGTGAG	58.3	19–976	8.82	97	97	100	0.351	0.366	0.351	0.370
		R: CAACTCTGCATGAATAAC										
CsCAT16	TC	F: CTCCTTGACTTTGAAGTTGC	58.3	18–977	9.55	105	105	100	0.355	0.366	0.355	0.375
		R: CTGATCGAGAGTAATAAAG										
EMCs38	AG	F: TTTCCCTATTTCTAGTTTGTGATG	60.0	20–976	9.73	107	107	100	0.405	0.355	0.405	0.393
		R: ATGGCGCTTTGGATGAAC										
Average					9.22	101.4	101.4	100	0.395	0.357	0.395	0.393
Total					46.10	507	507					

*Ta* annealing temperature, *SR* size range, *avNa* number of average alleles, *Na* number of total alleles, *Npa* number of polymorphic alleles, *P* polymorphism, *H*_*avp*_ mean heterozygosity, *PIC* polymorphism information content, *MI* marker index, *D* discriminating power.

The mean heterozygosity value (H_avp_) was determined to be the highest for the CsCAT1 marker (0.464) and the lowest for the CsCAT6 marker (0.351) (avg. 0.395 for all cultivars) (Table 1). Since we used iMEC indices in our study, the observed heterozygosity (Ho) value, as the closest criterion, was taken into account to compare the H_avp_ value. Different values have been previously obtained among studies in which the Ho value was analysed. For example, in Janfaza et al.^39^, the Ho value was determined to be 0.563 on average, while the expected heterozygosity (He) value was determined to be 0.512. In Martin et al.^33^, the highest Ho value, obtained with the CsCAT3 marker, was 0.906. According to Mattioni et al.^31^, the Ho obtained with the help of different SSR markers (not used in this study) was determined to be 0.52, 0.64 and 0.57 in chestnuts taken from the eastern (5 genotypes), western (5 genotypes) and central (5 genotypes) parts of Türkiye, respectively. In another study, Ho values were determined to be 0.78 and 0.73^36^.

The highest polymorphism information content (PIC) values were detected in the CsCAT6 (0.366) and CsCAT16 (0.366) markers, while the lowest PIC value was detected in the CsCAT1 marker, with a value of 0.339 (avg. 0.357 for all cultivars) (Table 1). In a previous study, genetic relationship analysis in Chinese chestnuts (*Castanea mollissima* Blume)^28^ was performed using 18 fluorescently labelled SSR markers produced from 146 chestnut genotypes, and the mean PIC value per locus was determined to be 0.622, a higher PIC value than that obtained in our study. In our study, the marker index (MI) was determined to be between 0.351 (CsCAT6) and 0.464 (CsCAT1) (avg. 0.395) (Table 1). Additionally, the discriminating power (D) recorded was between 0.370 (CsCAT16) and 0.435 (CsCAT3) (avg. 0.393) (Table 1).

**Figure 1:**
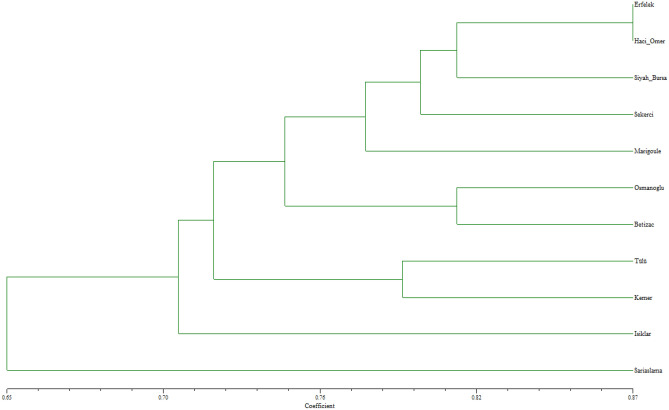
UPGMA dendrogram generated with genomic SSR markers in chestnut cultivars.

**Figure 2:**
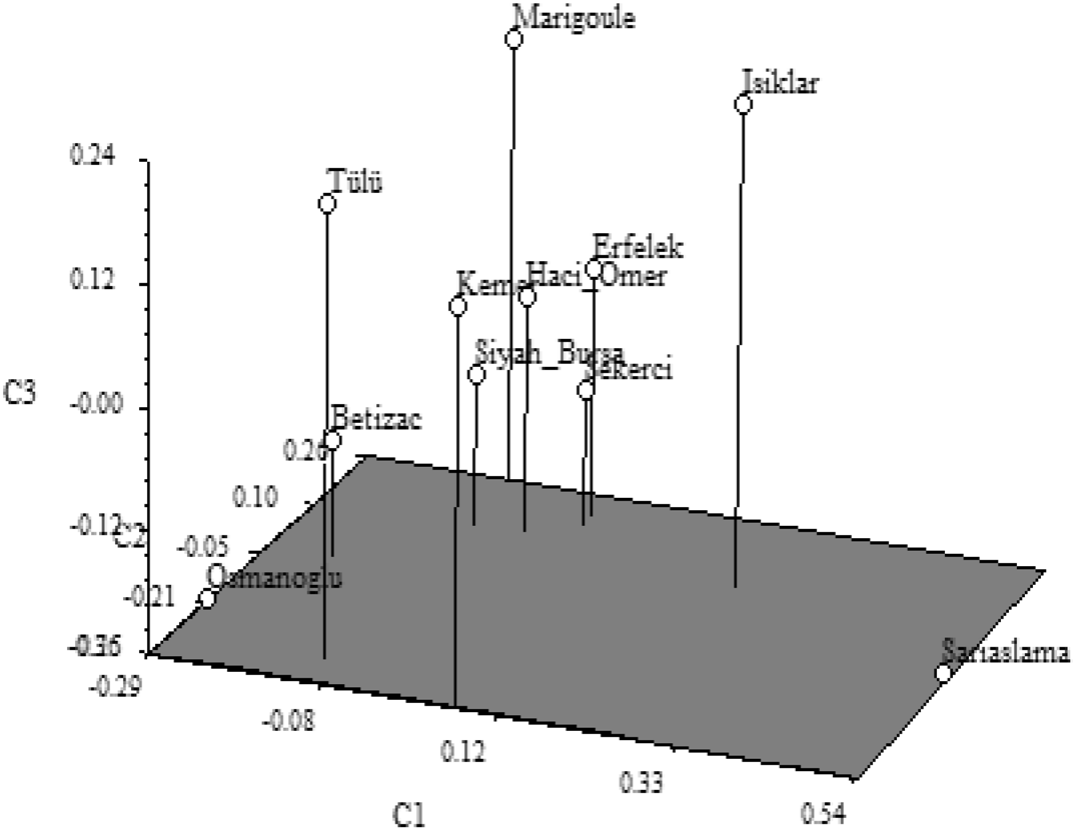
PCoA analysis with genomic SSR markers for chestnut cultivars.

The dendrogram resulting from the analysis of the data from the genomic SSR markers used in our study (Fig. 1) identified a group of closely related (similar) chestnut cultivars. In the dendrogram, the Erfelek and Hacıömer cultivars were determined to be the most similar cultivars, with a similarity coefficient value of 0.873. The cultivars showing close similarity with these two cultivars were Siyah Bursa, Şekerci and Marigoule, while the cultivars Osmanoğlu and Bouche de Betizac, which have a similarity coefficient of 0.810 with these cultivars, were determined to be closely related, and all these cultivars emerged as a subgroup. The Tülü and Kemer cultivars with a similarity coefficient of 0.790, were included in the other subgroup. When these two subgroups were evaluated as a group, the Işıklar and Sarıaşlama cultivars were found to be more distantly related to the cultivars in this group according to the dendrogram. The PCoA, which was performed to determine the genetic relationships as a result of SSR analyses, is given in Fig. 2. According to this analysis, the genetic distances among chestnut cultivars were found to support the data obtained from the dendrogram.

### Genetic relationship evaluation using EST-SSR markers

SSR markers derived from expressed sequence tags (EST) are increasingly used for the assessment of genetic variation and for more effective preservation of tree-form genotypes. They are developed from expressed regions of the genome with known functions. Numerous ESTs are available for many plant species and include EST-SSR markers^22,25,46,47^. Although they have been reported to be less polymorphic than genomic SSRs, EST-SSR markers are considered to have superior functional diversity with regard to adaptive variation and interspecies transferability^22,25,48,49^.

Using eight genetic EST-SSR markers in this study, the genetic relationship among chestnut cultivars was successfully determined, and the similarities and differences could also be determined. The *Na* and *Npa* values for a total of 728 alleles from the use of eight primers (avg. 91 alleles per primer) were found to be the same. Therefore, the *P* was determined to be 100% (Table 2). The EST-SSR markers that gave the highest Na and Npa were FIR059 (115 alleles) and VIT057 (102 alleles), while the EST-SSR marker that gave the lowest Na and Npa was GOT021 (74 alleles) (Table 2). In addition, the *avNa* values for each EST-SSR marker were determined to be 8.27 (FIR080), 7.73 (GOT004), 6.73 (GOT021), 9.27 (VIT057), 10.45 (FIR059), 5.55 (FIR094), 7.45 (GOT045) and 6.54 (VIT033) (Table 2). However, the same EST-SSR markers were used in chestnuts by Alcaide et al.^22^, and 14 (FIR059), 6 (FIR080), 4 (FIR094), 3 (GOT004), 4 (GOT021), 4 (GOT045), 2 (VIT033) and 2 (VIT057) alleles were detected. They identified two specific alleles with marker FIR059 and one specific allele with marker GOT021 associated with drought tolerance. In the same study, the highest number of effective alleles (Ne) was obtained from the FIR059 (6.59) and GOT021 (2.33) markers.

**Table 2:** Some parametres obtained with genic EST-SSR markers for eleven chestnut cultivars.

EST-SSR loci	Locus motif	Forward/reverse primer sequences (5′ → 3′)	Ta (°C)	SR (bp)	avNa	Na	Npa	P (%)	H_avp_	PIC	MI	D
FIR059	GA	F: GGTGGTTTCCGTGAGCATAG	57.2	23–976	10.45	115	115	100	0.377	0.240	0.377	0.304
		R: TTGCCACACCTTCTCGTTAG										
FIR080	ACC	F: ACCATACCTGGCTTCGATGA	52.6	21–975	8.27	91	91	100	0.240	0.281	0.240	0.980
		R: AAGGTGAGTTGGTGGTGGAG										
FIR094	CT	F: CAAAAGCCTCTCACTCTTGAGC	60.0	19–975	5.55	94	94	100	0.248	0.278	0.248	0.979
		R: TCAAACCCAAACAAAACGAA										
GOT004	TG	F: GGGCATATTGATCGCTTAGG	60.0	17–976	7.73	85	85	100	0.343	0.250	0.343	0.952
		R: TGAGCATTCATACATTCCATGAT										
GOT021	AT	F: AGAAAGTTCCAGGGAAAGCA	57.2	19–972	6.73	74	74	100	0.326	0.256	0.326	0.958
		R: CTTCGTCCCCAGTTGAATGT										
GOT045	CT complex	F: TCAACAAAACCCATTAAACCAA	54.0	19–973	7.45	82	82	100	0.331	0.254	0.331	0.956
		R: GGATCGGAGTGAAATGGAGA										
VIT057	AACTCG	F: TCAGCAAAATCCCAACTTTGT	54.0	20–976	9.27	102	102	100	0.343	0.250	0.343	0.951
		R: ACACTTCGCTGTTCCTCGAT										
VIT033	(CTT)(CCT)CTT	F: CATGAAGAACACACACGATGC	54.0	17–976	6.54	86	86	100	0.301	0.264	0.301	0.966
		R: TTCGGTGAACTTGAACTAGGC										
Average					7.75	91	91	100	0.309	0.262	0.309	0.287
Total					61.99	728	728					

*Ta* annealing temperature, *SR* size range, *avNa* number of average alleles, *Na* number of total alleles, *Npa* number of polymorphic alleles, *P* polymorphism, *H*_*avp*_ mean heterozygosity, *PIC* polymorphism information content, *MI* marker index, *D* discriminating power.

In our study, the *H*_*avp*_ was determined to be between 0.240 (FIR080) and 0.377 (FIR059) (avg. 0.309 for all cultivars) (Table 2). However, the highest Ho values determined by Alcaide et al.^22^ were 0.699 and 0.691 from the FIR059 and GOT021 markers, respectively. Moreover, in our study, the *PIC, MI* and *D* values were determined to be between 0.240 (FIR059)-0.281 (FIR080), 0.240 (FIR080)-0.464 (FIR059), and 0.304 (GOT021)-0.980 (FIR059), respectively (Table 2).

**Figure 3:**
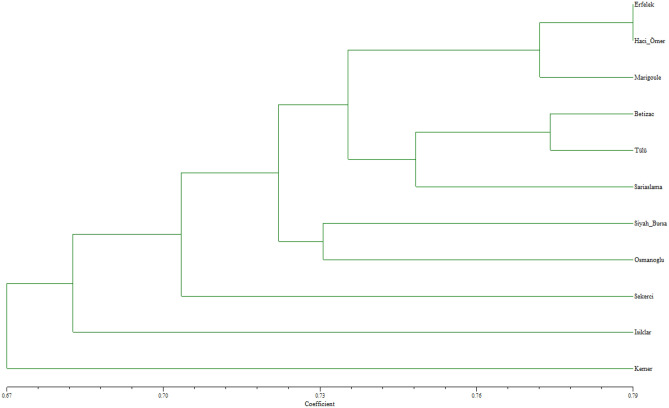
UPGMA dendrogram generated with genic EST-SSR markers in chestnut cultivars.

**Figure 4:**
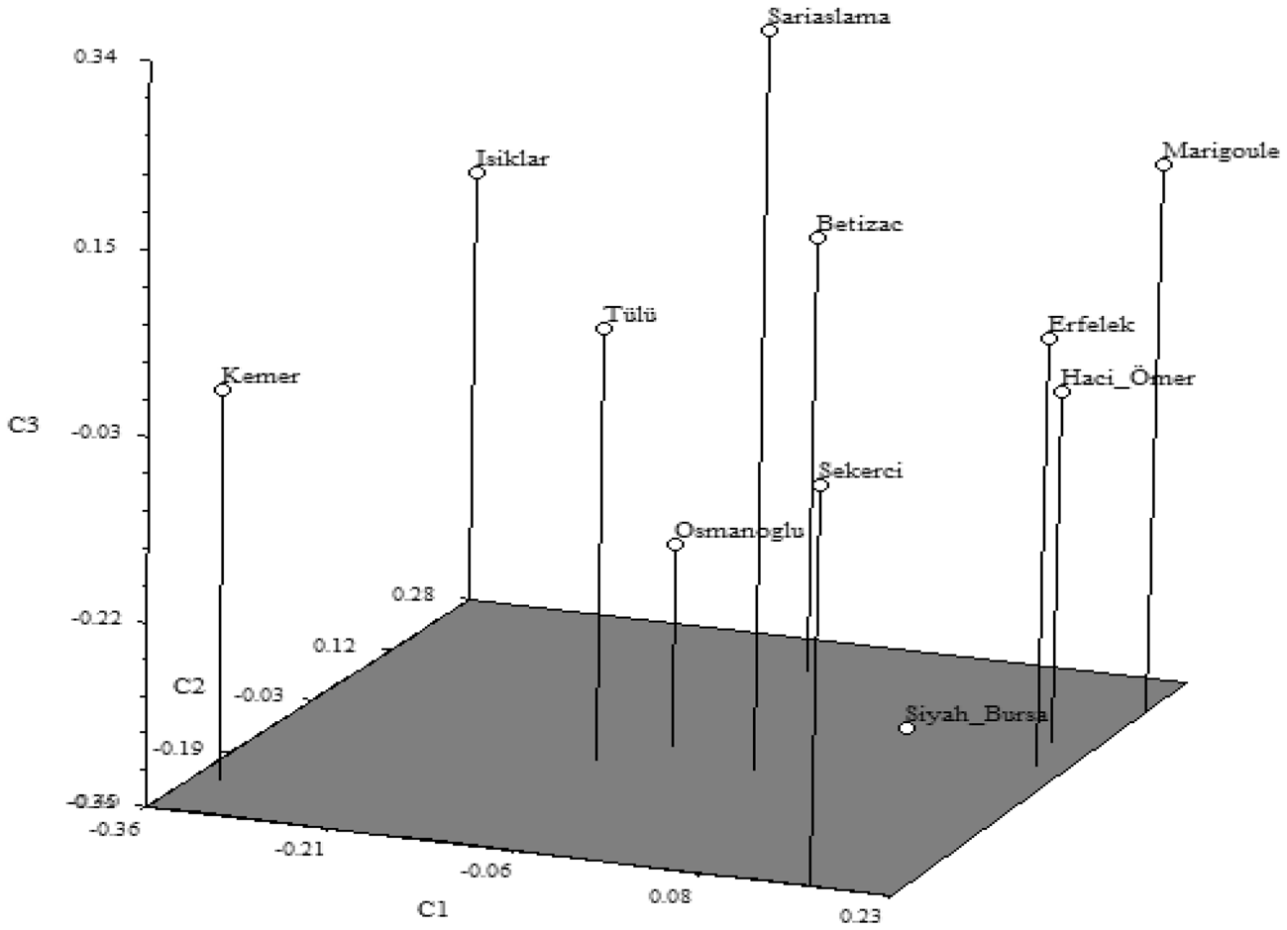
PCoA analysis with genic EST-SSR markers for chestnut cultivars.

According to the dendrogram obtained with drought-related genetic EST-SSR markers (Fig. 3), the Erfelek and Hacıömer chestnut cultivars were the most similar cultivars (0.789 similarity coefficient). Together with the Marigoule cultivar, which is closely related to these two cultivars, they all formed a subgroup. The Bouche de Betizac, Tülü and Sarıaşlama cultivars were closely related to this subgroup and formed another subgroup. However, the cultivars Siyah Bursa, Osmanoğlu, Şekerci, Işıklar and Kemer were found to be more distantly related to these groups. The PCoA results obtained with EST-SSR markers showing the genetic variation among chestnut cultivars is given in Fig. 4. According to this PCoA, the genetic relationship among chestnut cultivars supported the results obtained from the dendrogram.

The genetic similarities and differences among cultivars in both dendrograms (Figs. 1 and 3) may be due to the difference in the number of genomic SSR and EST-SSR markers used and to the different parts of the genome amplified by the two marker types^50,51^.

### Tolerance evaluation of chestnut cultivars based on drought-related EST-SSR markers

Assessing drought tolerance can be challenging. Drought tolerance is genetically polygenic and is associated with a complex expression pattern of dehydration-inducible genes^52^. Additionally, drought tolerance includes mechanisms operating at different spatial and temporal scales^53^.

It is estimated that climate change will cause longer summer drought periods and increase the frequency and severity of drought events in Mediterranean-type ecosystems where Türkiye is located. In a study by Alcaide et al.^22^, eight drought-related EST-SSR markers developed from oak species (*Quercus* spp.)^54^ were used to investigate the potential for drought tolerance adaptation of four wild populations of *C. sativa*. Among the markers used, the FIR059 marker showed three specific alleles (143, 160 and 179 bp) for drought-susceptible individuals and two specific alleles (152 and 176 bp) for drought-tolerant individuals (Table 3). The researchers found that these markers can be used as candidate markers to predict drought tolerance in chestnut species. Additionally, the FIR059 marker can be used in marker-assisted selection stages to predict drought tolerance in *C. sativa* trees. That study also identified one specific allele for drought-tolerant individuals (94 bp) and one specific allele for drought-susceptible individuals (97 bp) with the GOT021 marker obtained with the ABI-PRISM-3130XL genetic analyser. Additionally, a specific allele (193 bp) for drought-tolerant individuals was identified with the FIR094 marker and a specific allele (152 bp) for drought-susceptible individuals was identified with the FIR080 marker (Table 3). Castellana et al.^55^ carried out a genetic diversity study using microsatellite (SSR) markers based on the knowledge that climatic changes in chestnut growing areas in Europe cause sensitivity in chestnuts^56^ and on the knowledge of adaptability in natural habitats for this species. In their study, five genomic SSRs and eight genic EST-SSR markers were used, in addition to 268 genotypes belonging to 10 European chestnut populations from regions with different climatic characteristics. A total of 202 associations were identified among 22 different alleles, 9% of which were associated with the FIR059 locus. The results underline the close relationship between climate and genetic variability and show how this approach can provide valuable information for forest species management in a rapidly changing environment. The relationships between climate variables and genetic variation could be determined with drought-related markers in chestnuts obtained from Spain, Italy, Greece and Türkiye. Successful results were obtained with the use of SSR and EST-SSR markers used by these researchers (and preferred in our study) for chestnuts from two different climatic regions of Türkiye (a region with low precipitation-high temperature and a region with heavy precipitation-low temperature)^55^. For this reason, instead of developing new markers, these developed character-specific markers were used as the basis in our study. EST-SSRs related to drought stress have been reported in oak (*Quercus* spp.)^54,57,58^, chestnut (*Castanea* spp.)^33^ and walnut (*Juglans* spp.)^59^. Furthermore, drought-associated SSR markers^27,37^ were successfully used for chestnut genotypes.

**Table 3:** Specific alleles (bp) associated with drought tolerance identified in this study and identified in previous studies for chestnut cultivars.

EST-SSR markers	Specific alleles identified by Castellana et al.^55^	Specific alleles identified by Alcaide et al.^22^		Data of our study	
		Drought tolerance	Drought susceptibility	Specific alleles from this study	Cultivars
FIR094	185, 193, 197	193	–	48, 62	Erfelek, Hacıömer, Marigoule, Sarıaşlama, Bouche de Betizac, Işıklar
GOT045	137, 143	–	–	26, 30, 52, 66, 74	Erfelek, Hacıömer, Marigoule, Sarıaşlama
				26, 30	Şekerci
GOT021	99, 113	94	97	89, 110	Erfelek
				90, 112	Işıklar
VIT033	79, 81	–	–	51, 58	Hacıömer, Marigoule
				57	Bouche de Betizac, Işıklar, Tülü, Kemer
				52	Siyah Bursa, Şekerci
GOT004	–	–	–	52, 64, 82	Marigoule, Osmanoğlu, Tülü
				51, 63, 83	Sarıaşlama, Bouche de Betizac
				51, 63	Işıklar, Kemer
FIR059	181	152, 176	143, 160, 179	Specific allele could not be identified	
FIR080	144, 148	–	152	Specific allele could not be identified	
VIT057	–	–	–	Specific allele could not be identified	

In our study, a capillary electrophoresis (Qiagen-QIAxcel Advanced) system was used. Different allele sizes were detected compared to the allele sizes determined in previous studies also in chestnuts. Moreover, as seen in Table 3, there were differences in terms of the specific alleles obtained in the analysis carried out by Castellena et al.^55^ and Alcaide et al.^22^. The reasons for this can be very different. For example, Mattioni et al.^31^ noted that "*a genetic difference has emerged between eastern (Greek and Turkish) and western (Italian and Spanish) chestnut populations*". The differences in genetic diversity levels, observed with regional and geographic specificity in chestnuts, may be why specific alleles linked to drought-related tolerance by those researchers, were not detected in our study.

**Figure 5:**
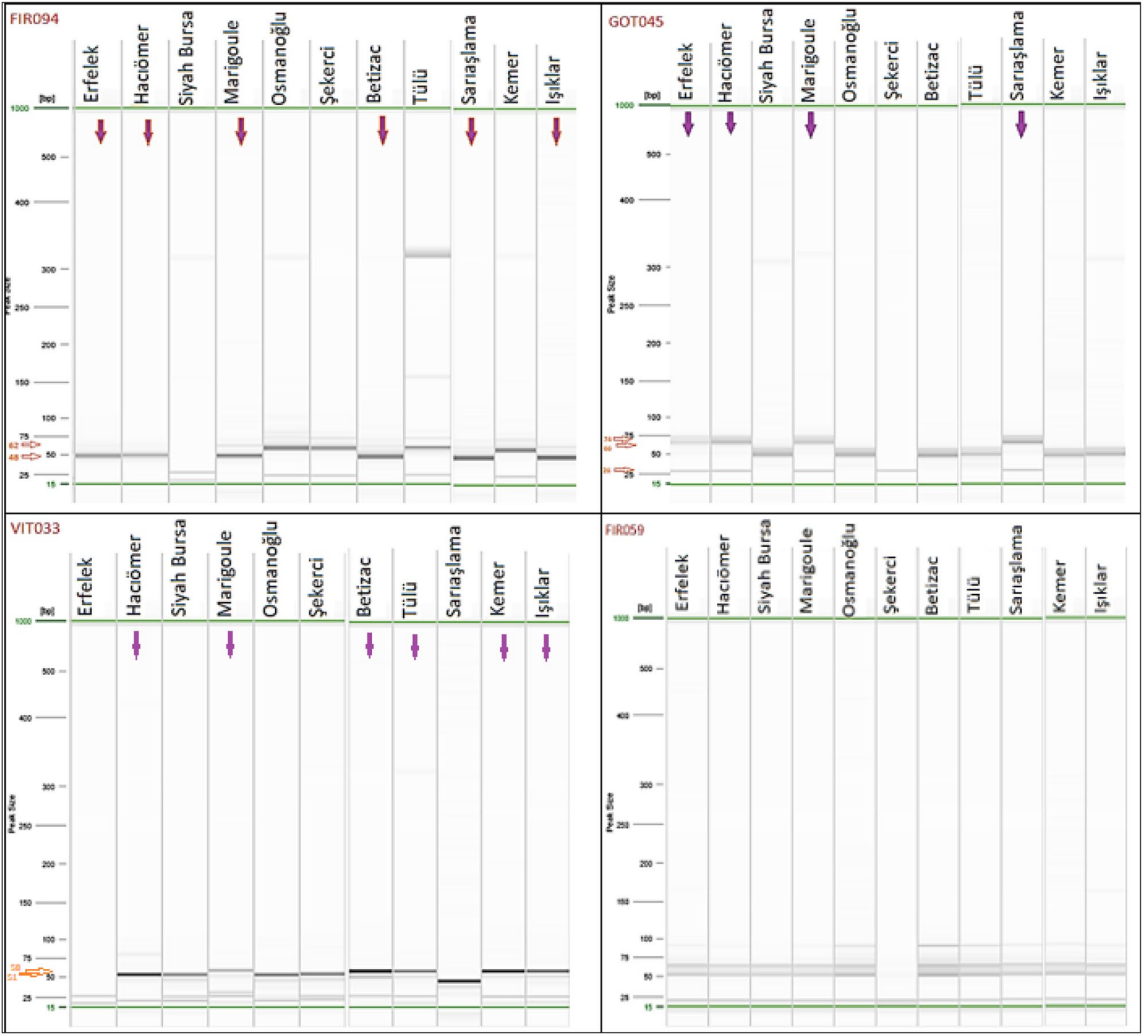
Some drought tolerance assessments for chestnut cultivars on capillary electrophoresis images obtained using primers FIR094, GOT045, VIT033 and FIR059 (the arrows indicate similar allele results).

According to the peak values and images obtained in the capillary system using genic EST-SSR markers, some assessments in terms of drought tolerance among the chestnut cultivars used in our study are listed below:

- With the **GOT045** marker, the chestnut cultivars Erfelek, Hacıömer, Marigoule, Sarıaşlama (26, 30, 52 and 66 bp) and Şekerci (26 and 30 bp) were found to have certain common alleles and therefore are closely related (Table 3, Fig. 5). Serdar et al.^10^ and Serdar and Macit^11^ reported that the Marigoule chestnut cultivar was drought-resistant. Based on this information, the Erfelek, Hacıömer, Sarıaşlama and Şekerci cultivars, similar to the Marigoule cultivar, are likely drought-tolerant chestnut cultivars (Table 3, Fig. 5).
- Based on the 48 and 62 bp alleles determined by the **FIR094** marker in our study, the chestnut cultivars Erfelek, Hacıömer, Marigoule, Sarıaşlama, Bouche de Betizac and Işıklar were to be closely related (Table 3, Fig. 5).
- According to the result of the **VIT033** marker, which is shown as a strong drought-tolerance marker^55,60^, the cultivars Siyah Bursa and Şekerci (52 bp), the cultivars Bouche de Betizac, Tülü, Kemer and Işıklar (57 bp), and the cultivars Hacıömer and Marigoule (51, 58 bp) were identified to be closely related to each other (Table 3, Fig. 5).
- In Castellana et al.^55^, the expected specific allele size for the **GOT021** marker was 113 bp. In our study, a 112-bp allele was obtained in the Işıklar cultivar with the same marker. In addition, for the GOT021 marker, the 94-bp allele was reported to be associated with drought tolerance in Alcaide et al.^22^, and the 90-bp allele observed in the Işıklar cultivar in the electrophoresis image obtained with the GOT021 marker in our study may be associated with drought tolerance. Again, the 89-bp and 110-bp alleles observed in the Erfelek cultivar in the electrophoresis image obtained with the GOT021 marker may be associated with drought tolerance (Table 3).
- The 52-, 64- and 82-bp alleles observed with the marker **GOT004** were determined in the chestnut cultivars Osmanoğlu, Marigoule, Sarıaşlama, Bouche de Betizac Tülü, Kemer, and Işıklar (Table 3).
- Although images were obtained from the **FIR059** (Table 3, Fig. 5), **FIR080** and **VIT057** (Table 3) markers in our study, these markers are not specific enough to comment on the relationship with drought.

## Conclusions

The microsatellite-based SSR and EST-SSR markers used in our study are specific markers developed from drought-related genes. Since source information was available that only the Marigoule cultivar was drought-tolerant among the 11 chestnut cultivars, clear interpretations could not be made in evaluating the results obtained with the SSR and EST-SSR markers used. Moreover, in previous studies using these markers, very different alleles were obtained from the allele information determined for tolerance and sensitivity. For these reasons, further studies are needed to confirm the accuracy of the molecular characterization results of the chestnut cultivars obtained in this study. For this purpose, there is a need for comprehensive evaluations that will include (i) the preliminary determination results obtained in this study, (ii) chestnut genotypes from Türkiye's genetic resources, and (iii) cultivars with correct names from chestnut collection gardens in Agricultural Research Institutes in Türkiye. Additionally, information that will contribute to chestnut breeding studies can be obtained by identifying a few key markers that will facilitate the determination of drought tolerance and incorporating this information into climatic data.

Türkiye is rich in chestnut germplasm resources. Although the microsatellite markers used in this study are drought-specific markers, character-specific markers can also be used in genetic diversity and characterization studies. If these markers are included in chestnut breeding studies, they can also contribute to the effective planning of the breeding process, such as genetic relatedness levels in chestnuts and rootstock-scion selection.

As a result of our study, the genetic relationships among chestnut cultivars that are of great importance in Türkiye could be characterized with drought-related character-specific markers. Moreover, although additional research is needed, some preliminary results regarding drought tolerance in chestnut cultivars have been obtained with the help of these markers.

## Materials and methods

### Plant materials

A total of eleven chestnut cultivars were used, which are the most commonly used in Türkiye and have high commercial value. These chestnut cultivars were obtained from a certified propagation nursery and were correctly labelled using their respective cultivar names. Nine chestnut cultivars (*C. sativa* Mill.) originating in Türkiye named Erfelek, Hacıömer, Osmanoğlu, Siyah Bursa, Şekerci, Sarıaşlama, Kemer, Işıklar, and Tülü were included. In addition to these cultivars, two hybrid chestnut cultivars of French origin (*C. sativa* × *C. crenata*) named Marigoule and Bouche de Betizac were also used in the study. Young leaf samples in the scion part of grafted chestnut plant materials were used (April–May 2022). The plant materials were transported to the laboratory under cold chain conditions and stored in a deep freezer (− 80 °C).

### DNA isolation and analysis

The DNA isolation protocol modified from Li and Quiros^61^ was used. For each sample, 0.3 g of plant material was digested with liquid nitrogen and prepared for DNA isolation. Then, 1000 µl of DNA extraction buffer (including 0.2% β-mercaptoethanol and 0.2 g PVP) was added to the plant material in a 2-ml tube. The tubes were incubated at 65 °C for 60 min. After incubation, 0.4 ml of chloroform was added to the tubes and centrifuged at 14,000 rpm for 20 min (+ 4 °C). The supernatant was transferred to a new tube. Then, 2.5 µl of RNase and 8 µl of proteinase-K were added and incubated at 37 °C for 45 min. Then, 100 µl of ammonium acetate and 2.5 times the volume of isopropanol were added to each tube to precipitate the DNA. The tubes were centrifuged at 14,000 rpm for 20 min and then centrifuged again for 1 min. The DNA was washed with 70% ethanol and dissolved in 100 ml of 1 × TE buffer. We suspended the DNA at + 4 °C for 24 h and stored it at − 20 °C. The DNA quantity and purity of the samples were determined. For this, optical densitometric spectrophotometer (NanoDrop-1000) readings were made at 260 and 280 nm. The DNA concentrations were then equalized. The DNA concentration was prepared at 50 ng/µl, and the DNA was stored at -20 °C. For the qualitative determination of DNA samples, electrophoresis was performed at 60 V on a 0.8% (w/v) agarose gel prepared with 1 × TBE buffer.

### Polymerase chain reaction (PCR) analysis for SSR and EST-SSR markers

In this study, genomic SSR loci^55^ and genic EST-SSR loci^22,55^ were used to determine drought-related tolerance in chestnuts (Table 1). Gradient PCR was performed between 50–60 °C for SSR and EST-SSR analyses, and the annealing temperatures of the primers are given in Table 1. The PCR steps were performed in the following order: 3 min at 95 °C for pre-denaturation, then 3 s at 94 °C for denaturation, 1 min at 57 °C for annealing, 1 min at 72 °C for elongation (35 cycles) and a final step at 72 °C for 30 min. The PCR components used in addition to the QIAxcel DNA Kit content for all SSR primers were 1 µl of 10 pmol/µl primer, 8 µl Taq DNA polymerase (Amplicon), 5 µl ultrapure water, and 1 µl genomic DNA (50 ng/µl). Electrophoresis of PCR products was performed using the QIAxcel DNA Kit (with QX alignment marker and QX DNA size marker) on the Qiagen Capillary analysis system.

### Scoring and data analysis for SSR and EST-SSR markers

In this study, the images obtained from capillary electrophoresis were scored for all primers. The genetic similarity values among individuals were analysed using the Numerical Taxonomy and Multivariate Analysis System (NTSYS)^62^. NTSYS, a statistical programs used in data analysis, can be used for multivariate data. The matrix is created by using “1” for the presence of characters and “0” for the absence of characters in the variables. Dendrogram creation and principal component analysis (2D and 3D) can be performed in the program. Dendrograms are a representation of data divided into nested sections at various levels, and vertical lines represent clusters that come together. The position of the lines on the dendrograms indicates the distances among clusters when they come together. With cluster analysis, it is possible to group similar observations into a series of clusters based on the observed values of a large number of variables for each individual. The principal component analysis (PCA) method is an orthogonal (vertical) transformation that transforms a set of possible related variables into a set of linearly unrelated variables called principal components^63^. Principal coordinate analysis (PCoA) is a multidimensional scaling method used to identify and visualize the similarities or differences in data. PCoA is a generalization of PCA and involves measuring the similarity between variables. It starts with a similarity matrix or dissimilarity matrix (distance matrix) and assigns for each element a position in a low-dimensional space as a 3D graph^64^. The clustering analysis and similarity indices among individuals were calculated using the Jaccard^65^ similarity coefficient with the help of the unweighted pair group method with arithmetic average (UPGMA)^66^. The polymorphism rate for each primer was calculated with the following formula: Polymorphism (%) = (Number of polymorphic alleles/Number of total alleles) × 100. The polymorphism rate is a value expressed for cases where the prevalence rate of gene polymorphism in the population is ≥ 1%. It is determined by dividing the total number of polymorphic bands obtained from the primers by the total number of bands.

In addition, some indices of polymorphism were determined for each amplified primer using the iMEC online tool^67^. Among the iMEC indices, arithmetic mean heterozygosity (H_avp_) calculated for polymorphic markers is determined by the formula H_avp_ = Σ Hn/np (observed heterozygosity). The polymorphism information content (PIC) is used to measure the information level of a genetic marker. This value is calculated with the formula PIC = 1 − Σ pi^2^ − Σ Σ pi^2^ pj^2^. The PIC value is used to measure the information level of a genetic marker. It differs from heterozygosity and refers to the value of a marker for detecting polymorphism, depending on the number of alleles and the distribution of their frequencies. The discriminating power (D) is determined by the Formula D = 1 − C and is based on the distribution of alleles in the sampled individuals. The D value evaluates the effectiveness of primers in identifying genotypes. It is strongly associated with the ability to distinguish among analysed samples. The marker index (MI) is the product of the effective multiplex ratio and the mean heterozygosity for polymorphic markers and is determined by the formula MI = E H_avp_. The term ‘effective multiplex ratio (E)’ referred to herein is expressed as the number of polymorphic loci in the relevant germplasm set analysed per experimental fraction of polymorphic loci. All of these indices are based on dominant and co-dominant DNA fingerprint markers. These allow the comparison and selection of the most suitable genetic markers for a given dataset^68^.

### Guidelines statement

All methods were carried out in accordance with the relevant guidelines/regulations/legislation.

## Data Availability

All data generated or analysed during this study are included in this published article.
